# A Comparison of Microbial Genome Web Portals

**DOI:** 10.3389/fmicb.2019.00208

**Published:** 2019-02-22

**Authors:** Peter D. Karp, Natalia Ivanova, Markus Krummenacker, Nikos Kyrpides, Mario Latendresse, Peter Midford, Wai Kit Ong, Suzanne Paley, Rekha Seshadri

**Affiliations:** ^1^Bioinformatics Research Group, SRI International, Menlo Park, CA, United States; ^2^DOE Joint Genome Institute, Walnut Creek, CA, United States

**Keywords:** genome portals, microbial genomics, microbial genomes, genome databases, microbial genome databases

## Abstract

Microbial genome web portals have a broad range of capabilities that address a number of information-finding and analysis needs for scientists. This article compares the capabilities of the major microbial genome web portals to aid researchers in determining which portal(s) are best suited to their needs. We assessed both the bioinformatics tools and the data content of BioCyc, KEGG, Ensembl Bacteria, KBase, IMG, and PATRIC. For each portal, our assessment compared and tallied the available capabilities. The strengths of BioCyc include its genomic and metabolic tools, multi-search capabilities, table-based analysis tools, regulatory network tools and data, omics data analysis tools, breadth of data content, and large amount of curated data. The strengths of KEGG include its genomic and metabolic tools. The strengths of Ensembl Bacteria include its genomic tools and large number of genomes. The strengths of KBase include its genomic tools and metabolic models. The strengths of IMG include its genomic tools, multi-search capabilities, large number of genomes, table-based analysis tools, and breadth of data content. The strengths of PATRIC include its large number of genomes, table-based analysis tools, metabolic models, and breadth of data content.

## 1. Introduction

A number of web portals provide the scientific community with access to the thousands of microbial genomes that have been sequenced to date. This article compares the capabilities of the major microbial genome web portals to aid researchers in determining which portal(s) best serve their information-finding and analytical needs.

The power that a genome web portal provides to its users is a function of what data the portal contains, and of the types of software tools the portal provides to users for querying, visualizing, and analyzing the data. Query tools enable researchers to find what they are looking for. Visualization tools speed the understanding of the information that is found. Analysis tools enable extraction of new relationships from the data.

We assess the data content of each portal both according to the types of data it provides (e.g., does it provide regulatory network information, protein localization data, or Gene Ontology annotations?), and according to the number of genomes it provides. We assess the software tools provided by each portal in several major areas: genomics tools, metabolic tools, advanced search and analysis tools, web services, table-based analysis, and user accounts. Omics data analysis capabilities are also assessed, but are distributed among the preceding areas. In each area, we enumerate multiple software capabilities, such as the ability to paint omics data onto pathway diagrams. We must emphasize that many of the portals include a significant number of other capabilities that we consider to be outside the scope of a microbial-genome web portal, and that are therefore not within the purview of this study. The Results section examines the comparison criteria in detail; for a higher level summary of the results, see the Discussion section.

Search tools are a particularly important part of a portal because they determine the user's ability to find information of interest; therefore, we provide detailed comparisons of the search tools that each portal provides for finding genes, proteins, DNA and RNA sites, metabolites, and pathways. We call these multi-search tools because they enable the user to search multiple database (DB) fields in combination.

Although user friendliness is a critical aspect of any website, it is extremely difficult to assess objectively. We have assessed a small number of relatively objective user friendliness criteria, such as the types of user documentation available, the presence of explanatory tooltips (small information windows that appear when the user hovers over regions of the screen), and the speed of the site's gene page.

Our criteria for inclusion in the comparison were portals with a perceived high level of usage, large number of genomes, a relatively rich collection of tools, and sites that are actively maintained and developed. The portals we compare are BioCyc Caspi et al. (2018) (version 22.0, April 2018), KEGG Kanehisa et al. (2017) (version 87.1, August 2018), Ensembl Bacteria Kersey et al. (2018) (Release 40, July 2018), KBase Arkin et al. (2018) (versions during August 2018 to October 2018), IMG Chen et al. (2017) (version 5.0 August 2018), and PATRIC Wattam et al. (2014) (version 3.5.21, July 2018).

Related portals that are not included in this comparison are Entrez Genomes (whose capabilities are similar to Ensembl Bacteria), MicroScope Vallenet et al. (2017) (which uses Pathway Tools for its metabolic component and therefore has the same metabolic functionality as BioCyc), ModelSEED Henry et al. (2010) (which is a metabolic model portal, not a genome portal), the SEED Overbeek et al. (2014) (which has been inactive for a number of years and was subsumed by the PATRIC project), MicrobesOnline Dehal et al. (2010), iMicrobe (https://www.imicrobe.us/—a portal for metagenomes and transcriptomes, not for single genomes), and Microme (http://www.microme.eu/—the Microme website largely shut down as of January 2018).

### 1.1. Summary of the Portals

Here we introduce each portal. Note that some portals have some capabilities that are not covered in this comparison. For each portal we provide a hyperlink to a sample gene page.

#### BioCyc

BioCyc Caspi et al. (2016) and Karp et al. (2017) is a microbial genome web portal that integrates sequenced genomes with curated information from the biological literature, with information imported from other biological DBs, and with computational inferences. BioCyc data include metabolic pathways, regulatory networks, and gene essentiality data. BioCyc provides extensive query and visualization tools, as well as tools for omics data analysis, metabolic path searching, and for running metabolic models. We omit discussion of many BioCyc comparative genomics and metabolic operations under its Analysis → Comparative Analysis menu. Scientists can use the Pathway Tools software associated with BioCyc to perform metabolic reconstructions and create BioCyc-like DBs for in-house genome data.

BioCyc contains information curated from 89,500 publications. The curated information includes experimentally determined gene functions and Gene Ontology terms, experimentally studied metabolic pathways, and experimentally determined parameters such as enzyme kinetics data and enzyme activators and inhibitors. Curated information also includes textual mini-reviews that summarize information about genes, pathways, and regulation, with citations to the primary literature. The large amount of curated information within BioCyc is unique with respect to other genome portals.

Home page: https://biocyc.org/

Sample gene page (full): https://biocyc.org/gene?orgid=ECOLI&id=EG10823

Sample gene page (short): https://tinyurl.com/yd9pcwcq

Bulk download site: Available after licensing via https://biocyc.org/download.shtml.

#### KEGG

The Kyoto Encyclopedia of Genes and Genomes is a resource for understanding high-level functions of a biological system from molecular-level information. It includes a focus on data relevant for biomedical research (e.g., KEGG DISEASE and KEGG DRUG databases) and includes tools for analysis of large-scale molecular datasets generated by high-throughput experimental technologies.

Home page: https://www.kegg.jp/

Sample gene page (full): https://www.kegg.jp/dbget-bin/www_bget?eco:b2699

Sample gene page (short): https://tinyurl.com/yd8d9th8

Bulk download site: https://www.kegg.jp/kegg/download/

### Ensembl Bacteria

Ensembl Bacteria is a portal for bacterial and archaeal genomes. It does not have any data or tools for metabolism, pathways or compounds, focusing on genes and proteins. Its strengths seem to be in its large collection of gene and protein family data. Its capabilities are somewhat different from other Ensembl sites. In addition to BLAST, it includes a hidden Markov model (HMM) search tool for protein motifs. Pan-taxonomic comparative tools are available for key species. It also includes Ensembl's variant effect predictor, which can predict functional consequences of sequence variants.

Home page: https://bacteria.ensembl.org/

Sample gene page (full): https://bacteria.ensembl.org/Escherichia_coli_str_k_12_substr_mg1655/Gene/Summary?g=b2699;r=Chromosome:2822708-2823769;t=AAC75741;db=core

Sample gene page (short): https://tinyurl.com/ya8onsem

Bulk download site: https://bacteria.ensembl.org/info/website/ftp/index.html

#### KBase

KBase is an environment for systems biology research that provides more than 160 applications to support user-driven analysis of a variety of data ranging from raw reads to fully assembled and annotated genomes, and metabolic models. In addition to its genome-portal capabilities, KBase Arkin et al. (2016) enables users to assemble and annotate genomes, to analyze transcriptomics data, and to create metabolic models for organisms with sequenced genomes. Once a model is created, it can be analyzed using phylogenetic, expression analysis, and comparative tools. KBase also allows users to integrate custom code into their analysis pipeline and enables addition of external applications by their developers using a software development kit (SDK). Its other major aim is to support reproducible computational experiments, on models, that can be published and shared with other users.

Home page: https://kbase.us/

Sample gene page (full): https://narrative.kbase.us/#dataview/35926/2/1?sub=Feature&subid=b2699

Sample gene page (short): https://tinyurl.com/y8twmntz

Bulk download site: The KBase website says that a bulk download site is coming soon.

#### IMG

The Integrated Microbial Genomes (IMG) system is a resource for annotation and analysis of sequence data, integrated with environmental and other metadata to support genome and microbiome comparisons. In addition to being the vehicle for release of the data generated by the DOE Joint Genome Institute, it provides a suite of analytical and visualization tools available to explore and mine the data for biological inference. Custom data marts dedicated to specific research topics like synthesis of secondary metabolite (IMG-ABC) or viral eco-genomics (IMG/VR), are also included. Users can submit their own data and metadata for integration in the system.

Home page: https://img.jgi.doe.gov/

Sample gene page (full): https://img.jgi.doe.gov/cgi-bin/m/main.cgi?section=GeneDetail&page=geneDetail&gene_oid=646314661

Sample gene page (short): https://tinyurl.com/y988yzc9

Bulk download site: https://genome.jgi.doe.gov/portal/

#### PATRIC

PATRIC is designed to support the biomedical research community's work on bacterial infectious diseases via integration of vital pathogen information with data and analysis tools. Data is integrated across sources, data types, molecular entities, and organisms. Data types include genomics, transcriptomics, protein-protein interactions, 3D protein structures, sequence typing data, and metadata. It supports both genome assembly and annotation (RAST), and RNA-seq data analysis via a job submission system.

Home page: https://www.patricbrc.org/

Sample gene page (full):https://www.patricbrc.org/view/Feature/PATRIC.511145.12.NC_000913.CDS.2820730.2821791.rev

https://www.patricbrc.org/view/Feature/PATRIC.511145.12.NC_000913.CDS.2820730.2821791.rev

Sample gene page (short): https://tinyurl.com/ybkynwy9

Bulk download site: ftp://ftp.patricbrc.org/

## 2. Results

We assessed the software and data content capabilities of each portal according to a number of topic areas, such as genomics-related tools and metabolism-related tools. We chose topic areas that we considered to be core elements of a microbial genome information portal—that is, a web site that counts among its primary missions providing users with data and knowledge regarding sequenced microbial genomes. A number of the portals contain functionality outside of that mission, for example, some portals contain software tools for annotating microbial genomes (e.g., performing assembly and gene-function prediction). We did not include such functionality because we considered it outside the scope of a microbial genome information portal. In many cases, we added new criteria within a topic area (meaning rows within our comparison tables) as we learned about each portal, such as adding the ability of Ensembl Bacteria to predict the effects of sequence variants. Our choice of criteria is validated by the fact that many of the criteria are shared among some or many of the portals.

For several of the topic areas, we provide multiple tables to assess software capabilities, with one or two tables focusing on DB search capabilities and another table focusing on other capabilities in that area. For example, **Tables 2**, **3** describe genomics multi-search tools, and Table 1 describe other genomics software tools.

**Table 1:** Genomics tools comparison.

**Tool**	**BioCyc**	**KEGG**	**Ensembl Bacteria**	**KBase**	**IMG**	**PATRIC**
Genome browser	YES	YES	YES	YES	YES	YES
–Operons, promoters, TF binding sites	YES	no	no	no	Partial	YES
–Depicts nucleotide sequence	YES	YES	YES	YES	YES	YES
–Customizable tracks	YES	no	YES	no	Partial	YES
–Comparative, by orthologs	YES	no^a^	no	no	YES	YES
–Genome poster	YES	no	no	no	no	no
Retrieve gene sequence	YES	YES	YES	YES	YES	YES
Retrieve replicon sequence	YES	YES	YES	no	YES	YES
Retrieve protein sequence	YES	YES	YES	YES	YES	YES
Nucleotide sequence alignment viewer	YES	YES	no	no	YES	YES
Protein sequence alignment viewer	YES	YES	no	no	YES	YES
Protein phylogenetic tree analysis	no	YES	no	YES	YES	YES
Sequence searching by BLAST	YES	YES	YES	YES	YES	YES
Sequence pattern search	YES	YES	no	YES	YES	no
Sequence cassette search	no	YES	YES	YES	YES	no
Orthologs	YES	YES	no	YES	YES	YES
Gene/Protein page	YES	YES	YES	YES	YES	YES
Enrichment analysis (GO terms)	YES	no	no	YES	no	no
Enrichment analysis (regulation)	YES	no	no	no	no	no
Omics dashboard	YES	no	no	no	no	no
Multi-organism comparative analysis	YES	YES	YES	YES	YES	YES
Horizontal gene transfer prediction	no	no	no	no	YES	no
Fused protein prediction	no	no	no	no	YES	no
Alternative ORF view	no	no	no	no	YES	YES
Genome multi-search	YES	no	no	no	YES	YES
gANI computations	no	no	no	YES	YES	YES
Kmer frequency analysis	no	no	no	no	YES	no
Synteny comparison	no	no	no	YES	YES	no
Proteome comparisons	YES	no	no	YES	YES	YES
Statistical analysis, genome	YES	no	no	no	YES	no
Statistical analysis, expression	no	no	no	YES	YES	YES
Genome function comparison	no	no	no	YES	YES	YES
Insert genomes into reference trees	no	no	no	YES	no	YES^b^
Predict effects of sequence variants	no	no	YES	no	no	YES

“*Partial” means that the tool provides some but not all of the indicated functionality.*

a KEGG does have a rudimentary tool for this purpose, but it is not based on a zoomable genome browser.

b *PATRIC supports construction of trees from an arbitrary set of in-group and out-group genomes*.

### 2.1. Genomics Tools

Genomics tools enable researchers to query, analyze, and compare genome-related information within an organism DB. Table 1 assesses most genomics tools; Tables 2, 3 describe genomics multi-search tools.

**Table 2:** Gene/protein multi-search capabilities.

**Tool**	**BioCyc**	**KEGG**	**Ensembl Bacteria**	**KBase**	**IMG**	**PATRIC**
Gene name	YES	YES	YES	YES	YES	YES
Product name	YES	YES	YES	YES	YES	YES
Database identifier	YES	YES	YES	YES	YES	YES
EC number	YES	YES	YES	no	YES	YES
Sequence length	YES	no	no	YES	YES	YES
Replicon	YES	no	no	YES	YES	YES
Map position	YES	YES	no	YES	YES	no
Product mol wt	YES	no	no	no	YES	no
Product subunits	YES	no	no	no	YES	no
Product pI	YES	no	no	no	YES	no
Product ligands	YES	no	no	no	YES	no
Evidence code	YES	no	no	no	no	no
Cell component	YES	no	no	no	no	no
GO terms	YES	no	YES	YES	YES	YES
Protein features	YES	no	YES	no	YES	no
Publication	YES	no	no	YES	no	no
scaffold length	no	YES	no	YES	YES	no
Scaffold GC content	no	no	no	no	YES	YES
Protein family assignment	no	YES	YES	no	YES	YES
Is partial	no	no	no	no	YES	no
Is pseudogene	YES	no	no	no	YES	YES

*Does the portal support multi-searches for genes and gene products based on the data fields or criteria listed? “Publication” means the ability to search for a gene based on a publication cited in the pathway entry. “Scaffold Length” means the ability to search for a gene based on the length of the scaffold it resides on. “Protein Family Assignment” means the ability to search for a gene based on what protein families it is assigned to (e.g., Pfam or TIGRFAM family). “Is Partial” means search for partial (truncated) proteins*.

**Table 3:** DNA/RNA Site Multi-Search Capabilities.

**Tool**	**BioCyc**	**KEGG**	**Ensembl Bacteria**	**KBase**	**IMG**	**PATRIC**
Site type	YES	no	no	no	no	no
–Attenuators	YES	no	no	no	no	no
–Origin of replication	YES	no	no	no	no	no
–Phage attachment sites	YES	no	no	no	no	no
–REP elements	YES	no	no	no	no	no
–Promoters	YES	no	no	no	no	no
–Terminators	YES	no	no	no	no	no
–mRNA binding sites	YES	no	no	no	YES	no
–Riboswitches	YES	no	no	no	YES	no
–TF binding sites	YES	no	no	no	no	no
–Transcription units	YES	no	no	no	no	no
–Transposons	YES	no	no	no	no	no
Replicon	YES	no	no	no	YES	no
Map position	YES	no	no	no	YES	no
Site regulator	YES	no	no	no	no	no
Site ligands	YES	no	no	no	no	no
Evidence code	YES	no	no	no	no	no
CRISPR arrays	no	no	no	no	YES	no

Does the portal support multi-searches for DNA and RNA sites based on the data fields or criteria listed? For example, does the portal support searches for sites by the type of site (e.g., for attenuators vs. transcription-factor binding sites), and by numeric constraints on the genome position of the site?

An explanation of the rows within Table 1 is as follows.

- **Genome Browser**: Can a user browse a chromosome at different zoom levels to see the genomic features present?
  - Are **operons, promoters, and transcription-factor binding sites** depicted in the genome browser?
  - Is the **nucleotide sequence** depicted in the genome browser?
  - **Customizable Tracks**: Can a user add additional tracks to the genome browser, which show user-supplied data?
  - **Comparative, by Orthologs**: Can a user compare chromosome regions from several genomes side-by-side, with orthologous genes indicated?
  - **Genome Poster**: Can the portal generate a printable, detailed, wall-sized poster of the entire genome, e.g., one that depicts every gene in the genome?
- **Retrieve Gene Sequence**: Can a user retrieve the nucleotide sequence of a gene?
- **Retrieve Replicon Sequence**: Can a user retrieve the nucleotide sequence of a specified region of a replicon?
- **Retrieve Protein Sequence**: Can a user retrieve the amino-acid sequence of a protein?
- **Nucleotide Sequence Alignment Viewer**: Can a user compare the nucleotide sequence of a gene with orthologs from other organisms?
- **Protein Sequence Alignment Viewer**: Can a user compare the amino-acid sequence of a protein with orthologs from other organisms?
- **Protein Phylogenetic Tree Analysis**: Can a user construct a phylogenetic tree from a set of protein sequences?
- **Sequence Searching by BLAST**: Is searching for a sequence in a genome by BLAST supported?
- **Sequence Pattern Search**: Is sequence searching by short sequence patterns supported?
- **Sequence Cassette Search**: Is sequence searching by protein family recognition patterns supported?
- **Orthologs**: Can a user query for the orthologs of a given gene in other organisms?
- **Gene/Protein Page**: Does the portal provide gene pages, showing relevant information such as the gene products and links to other DBs?
- **Enrichment Analysis (GO Terms)**: Can a user find which GO terms are statistically enriched, given a set of genes?
- **Enrichment Analysis (Regulation)**: Given a set of genes, can a user compute which regulators of those genes are statistically over-represented in the gene set?
- **Omics Dashboard**: Can a user submit a transcriptomics dataset for analysis using a visual dashboard tool that enables interactive summarization and exploration of the dataset in a manner similar to the BioCyc Omics Dashboard Paley et al. (2017)?
- **Multi-Organism Comparative Analysis**: Can a user globally compare a variety of different data types between several organisms?
- **Horizontal Gene Transfer Prediction**: Can the site show which genes may have been acquired by horizontal gene transfer?
- **Fused Protein Prediction**: Can the portal show which genes result from fusions of genes that can be found separately in other organisms?
- **Alternative ORF Search (6-frame translation)**: Can a user assess alternative ORFs to the ones predicted on a given genomic region?
- **Genome Multi-Search**: Does the portal support search and retrieval across all genomes using sequencing, environmental, or other metadata attributes?
- **gANI (Whole-genome Average Nucleotide Identity) Computations**: Whole-genome based average nucleotide identity (gANI) has been proposed as a measure of genetic relatedness of a pair of genomes. gANI for a pair of genomes is calculated by averaging the nucleotide identities of orthologous genes. The fraction of orthologous genes (alignment fraction or AF) is also reported as a complementary measure of similarity of the two genomes.
- **Kmer Frequency Analysis**: Can the portal display principal component analysis plots of oligonucleotide frequencies along genome length; allow comparison of genomes by the similarity of oligonucleotide composition, and identify sequences with abnormal oligonucleotide composition, such as horizontally transferred sequences and contaminating contigs/scaffolds?
- **Synteny Comparisons**: Does the portal provide a tool for evaluating conservation of gene order by plotting pairwise genome alignment? Potential translocations, inversions, or gaps relative to reference can be visualized. Such a tool gives a quick snapshot of how closely related two strains might be.
- **Proteome Comparisons**: Find proteins that are shared between two or more genomes or unique to a given genome.
- **Statistical Analysis, Genome**: Example statistical analyses include counts of genes assigned to a “feature” (such as presence of a COG/Pfam/TIGRFAM/KEGG domains), and counts of genes in different Gene Ontology categories.
- **Statistical Analysis, Expression**: Does the portal provide tools for calculating statistical significance of gene expression data?
- **Genome Function Comparison**: Genomes can be clustered based on a function profile (e.g., COG/Pfam/TIGRFAM/KEGG features) and viewed as a hierarchical cluster tree, principal component analysis, principal coordinate analysis plot, or other options, to assess relatedness of selected genomes.
- **Insert Genomes into Reference Trees**: Enables a user to determine evolutionary relationships between a genome of interest and nearby reference genomes by building a tree of 49 concatenated universal sequences.
- **Predict Effects of Sequence Variants**: Enables users to predict effects of variation, including SNPs and indels on transcripts in the region of the variant.

### 2.2. Metabolic Tools

Metabolic tools enable researchers to query, analyze, and compare information about metabolic pathways and reactions within an organism DB, to run metabolic models, and to analyze high-throughput data in the context of metabolic networks. Table 4 assesses most metabolic tools; Table 5 describes metabolite multi-search capabilities and Table 6 describes pathway multi-search capabilities.

**Table 4:** Metabolic tools comparison.

**Tool**	**BioCyc**	**KEGG**	**Ensembl Bacteria**	**KBase**	**IMG**	**PATRIC**
Metabolite page	YES	YES	no	no	no	no
Chemical similarity search	no	YES	no	no	no	no
Glycan similarity search	no	YES	no	no	no	no
Reaction page	YES	YES	no	no	YES	no
–Reaction atom mappings	YES	YES	no	no	no	no
Individual pathway diagram	YES	YES	no	YES	YES	YES
–Automatic pathway layout	YES	no	no	no	no	no
–Paint omics data onto pathway	YES	YES	no	no	YES	no
–Depict enzyme regulation	YES	no	no	no	no	no
–Depict genetic regulation	YES	no	no	no	no	no
–Depict metabolite structures	YES	YES (Tooltip)	no	no	no	no
Multi-pathway diagram	YES	no	no	no	no	no
Full metabolic network diagram	YES	YES	no	no	no	no
–Zoomable metabolic network	YES	YES	no	no	no	no
–Paint omics data onto diagram	YES	no	no	no	no	no
–Animated omics data painting	YES	no	no	no	no	no
–Metabolic poster	YES	no	no	no	no	no
–Organism comparison	YES	no	no	no	no	no
Automated metabolic reconstruction	YES (Desktop)^a^	YES	no	YES	YES	YES
Enrichment analysis (Pathways)	YES	no	no	no	YES	no
Execute metabolic model	YES	no	no	YES	no	YES
–Gene knock-out analysis	YES	no	no	YES	no	YES
Chokepoint analysis	YES	no	no	no	no	no
Dead-end metabolite analysis	YES	no	no	no	no	no
Blocked-reaction analysis	YES	no	no	YES	no	no
Route search tool	YES	YES	no	no	no	no
Path prediction tool	no	YES	no	no	no	no
Assign EC number	no	YES	no	no	no	no

a *The desktop version of the Pathway Tools software performs automated metabolic reconstruction*.

**Table 5:** Compound multi-search capabilities.

**Tool**	**BioCyc**	**KEGG**	**Ensembl Bacteria**	**KBase**	**IMG**	**PATRIC**
Name	YES	YES	no	no	YES	YES^a^
Database identifier	YES	YES	no	no	YES	YES^a^
Ontology	YES	no	no	no	YES	YES
Monoisotopic mass	YES	no	no	no	Partial	no
Molecular weight	YES	no	no	no	Partial	no
Chemical formula	YES	no	no	no	Partial	no
Chemical substructure	YES	YES	no	no	Partial	no
InChi string	YES	no	no	no	Partial	no
InChi key	YES	no	no	no	Partial	no

Does the portal support multi-searches for chemical compounds based on the data fields or criteria listed? “Ontology” means the ability to search for compounds based on a chemical ontology (classification).

a *This search will find pages of antimicrobial compounds*.

**Table 6:** Pathway multi-search capabilities.

**Tool**	**BioCyc**	**KEGG**	**Ensembl Bacteria**	**KBase**	**IMG**	**PATRIC**
Name	YES	YES	no	no	YES	YES
Ontology	YES	YES	no	no	YES	YES
Size in reactions	YES	no	no	no	no	no
Substrates	YES	YES	no	no	YES	no
Evidence code	YES	no	no	no	no	no
Publication	YES	no	no	no	no	no

*Does the portal support multi-searches for pathways based on the data fields or criteria listed? “Ontology” means the ability to search for pathways based on a pathway ontology (classification)*.

An explanation of the rows within Table 4 is as follows.

- **Metabolite Page**: Does the site provide a metabolite page, showing relevant information such as synonyms, chemical structure, and reactions in which the metabolite occurs?
- **Chemical Similarity Search**: Can the user search for chemicals that have similar structures to a provided chemical?
- **Glycan Similarity Search**: Can the user search for glycans that have similar structures to a provided glycan?
- **Reaction Page**: Does the site provide a reaction page, showing relevant information such as EC numbers, reaction equation, and enzymes catalyzing the reaction?
- **Reaction Atom Mappings**: Can the reaction equation be shown with metabolite structures that depict the trajectories of atoms from reactants to products?
- **Pathway Diagrams**: Can pathway diagrams be depicted?
- **Automatic Pathway Layout**: Are pathway diagrams generated automatically by the software, thereby avoiding manual drawing?
- **Paint Omics Data onto Pathway**: Can a user visualize omics data on pathway diagrams?
- **Depict Enzyme Regulation**: Can pathway diagrams show regulation of enzymes by metabolites, to depict information such as feedback inhibition?
- **Depict Genetic Regulation**: Can pathway diagrams show genetic regulation of enzymes, such as by transcription factors and attenuation?
- **Depict Metabolite Structures**: Can pathway diagrams show the chemical structures of metabolites?
- **Multi-Pathway Diagram**: Can users interactively create diagrams consisting of multiple interacting metabolic pathways?
- **Full Metabolic Network Diagram**: Can the entire metabolic reaction network of a genome be depicted and explored by an interactive graphical interface?
- **Zoomable Metabolic Network**: Does the metabolic network browser enable zooming in and out?
- **Paint Omics Data onto Network**: Can a user visualize an omics dataset (e.g., gene expression, metabolomics) on the metabolic network diagram?
- **Animated Omics Data Painting**: Can several omics data points be visualized as an animation on the metabolic network diagram?
- **Metabolic Poster**: Can the portal generate a printable wall-sized poster of the organism's metabolic network?
- **Organism Comparison**: Can a user compare the metabolic networks of two organisms via the full metabolic network diagram?
- **Automated Metabolic Reconstruction**: Starting from a functionally annotated genome, can the metabolic reaction network (and pathways) be inferred in an automated fashion?
- **Enrichment Analysis (Pathways)**: Can the site compute statistical enrichment of pathways within a large-scale dataset?
- **Execute Metabolic Model**: Can a user execute a steady-state metabolic flux model via the portal?
- **Gene Knock-out Analysis**: Can a user run flux-balance analysis (FBA) on the metabolic network by systematically disabling (knocking-out) various genes, to investigate how knock-outs perturb the network, and to predict gene essentiality?
- **Chokepoint Analysis**: Can the site compute chokepoint reactions (possible drug targets) in the full metabolic reaction network? A chokepoint reaction is a reaction that either uniquely consumes a specific reactant or uniquely produces a specific product in the metabolic network.
- **Dead-End Metabolite Analysis**: Can the portal compute dead-end metabolites in the full metabolic reaction network? Dead-end metabolites are those that are either only consumed, or only produced, by the reactions within a given cellular compartment, including transport reactions.
- **Blocked-Reaction Analysis**: Can the portal compute blocked reactions in the full metabolic reaction network? Blocked reactions cannot carry flux because of dead-end metabolites upstream or downstream of the reactions.
- **Route Search Tool**: Given a starting and an ending metabolite, can the site compute an optimal series of known reactions (routes) that converts the starting metabolite to the ending metabolite?
- **Path Prediction Tool**: Given a starting chemical compound, can the site predict a series of previously unknown enzyme-catalyzed reactions that will act upon the input compound and the products of previous reactions?
- **Assign EC Number**: Can the portal compute an appropriate Enzyme Commission number for a user-provided reaction?

### 2.3. Regulation Tools

BioCyc has a number of regulatory informatics tools that are not provided by any of the portals. We list those tools here rather than providing a table.

- BioCyc includes a regulatory-network browser that depicts the full transcriptional regulatory network of the organism. The network diagram can be queried interactively and painted with transcriptomics data.
- The BioCyc transcription-unit page depicts operon structure including promoters, transcription factor binding sites, and terminators, the evidence for each, and describes regulatory interactions between these sites and associated transcription factors and small RNA regulators.
- BioCyc generates diagrams that summarize all regulatory influences on a gene, including regulation of transcription, translation, and of the gene product.
- BioCyc depicts transcription-factor regulons as diagrams of all operons regulated by a transcription factor.
- BioCyc can depict regulatory influences on metabolism by highlighting the regulon of a transcription factor on the BioCyc metabolic map diagram.
- BioCyc SmartTables can list the regulators or regulatees of each gene within a SmartTable.
- BioCyc can generate a report comparing the regulatory networks of two or more organisms.

### 2.4. Advanced Search and Analysis

These tools (see Table 7) enable researchers to perform complex searches and analyses, to retrieve data via web services and bulk downloads, and to create and manipulate user accounts.

**Table 7:** Comparison of advanced search and analysis, web Services, and user accounts.

**Tool**	**BioCyc**	**KEGG**	**Ensembl Bacteria**	**KBase**	**IMG**	**PATRIC**
Advanced search	YES	no	no	no	YES	no
Cross-organism search	YES	YES	YES	Partial	YES	YES
web services	YES	YES	YES	YES	no	no
Other query options	*	*	*	*	*	*
User account	Opt/req	no	Optional	Required	Opt/req	Opt/req
Custom notifications	YES	no	no	no	no	no
Download formats	Biopax,gff	Json,sbml	Fasta,gff,gff3	Genbank,gff,tsv	Fasta,txt	Csv,fasta,gff
	genbank		json,mysql,rdf	fasta,json,sbml		embl,json
	sbml					genbank

“*Opt/Req” means that user accounts are optional for some operations and required for other operations. IMG also provides for downloading of reads, assemblies, QC reports, annotations, and more*.

An explanation of the rows within Table 7 is as follows.

- **Advanced Search**: Does the site enable the user to construct multi-criteria queries that search arbitrary DB fields using combinations of AND, OR, and NOT?
- **Cross-Organism Search**: Can a user search all organisms, specified organism sets, or taxonomic groups of organisms, for genes, metabolites, or pathways?
- **Web Services**: Can DBs within the portal be queried programmatically by means of web services, using for example XML protocols?
- **Other Query Options:** What other query options are provided by the portal?
  - BioCyc supports queries via its BioVelo query language^1^. Users can download BioCyc data files for text searches, and can load those data files into a locally installed version of SRI's BioWarehouse system for SQL query access. Users can download bundled versions of subsets of BioCyc plus Pathway Tools, and query the DBs via APIs for Python, Lisp, Java, Perl, and R.
  - Users can download KEGG data files for text searches.
  - Ensembl Bacteria provides a Perl API and public MySQL servers.
  - KBase includes code cells for adding python code blocks to enable custom analyses, for which applications do not exist, or for programmatically calling Kbase native apps to automate large scale analyses.
  - PATRIC provides a downloadable command line interpreter application that allows interactive submission of DB queries using a query language.
- **User Account**: Are user accounts available for logging in, and for storing data and preferences? “Opt/Req” means accounts are optional for some operations and required for other operations.
- **Custom Notifications**: Does the portal enable the user to register to be notified of curation updates in biological areas of interest to the user?
- **Bulk Download Formats**: What formats are supported by the portal for large scale data downloads? The websites for bulk downloads are provided in section 1.1.

### 2.5. Table-Based Analysis Tools

Table-based analysis tools enable users to define lists of genes, proteins, metabolites, or pathways that are stored within the portal, and can be displayed, analyzed, manipulated, and shared with other users. These tools are called SmartTables by BioCyc and are called Carts by IMG. A typical series of SmartTable operations are to define a SmartTable containing a list of genes (such as from a transcriptomics experiment); to configure which DB properties are displayed for each gene within the SmartTable (such as displaying the gene name, accession number, product name, and genome map position); performing a set operation on the SmartTable such as taking the intersection with another gene SmartTable; and transforming the gene SmartTable to say a SmartTable of the metabolic pathways containing those genes, or the set of transcriptional regulators for those genes.

KBase does not have a tables mechanism, but it does have a data sharing mechanism called narratives, which is not table-based.

Table-based capabilities are summarized within Table 8; an explanation of its rows is as follows.

- **Datatypes Tables can Contain**: What types of entities may be stored in tables within each portal? The more types of entities can be manipulated within tables, the more versatile the table mechanism is.
- **Create Table from Uploaded File**: Can tables be defined by uploading a data file that lists the entities within the table?
- **Create Table from DB Query Result**: Can tables be defined from the result of a query within the portal?
- **Include DB Properties as Table Columns**: Can a user add columns to the table containing information from the DB about a given entity, such as the accession number of a gene or the nucleotide coordinate of a gene, or a diagram of the chemical structure of a metabolite?
- **Create Table Columns as Computational Transformations**: Can table columns contained information computed from another column, such as adding a column that computes the pathways in which a gene participates?
- **Set Operations Among Tables**: Can the portal create a new table by computing set operations between two other tables, such as taking the union of the list of genes in two other tables?
- **Filter Table Rows**: Can the portal remove rows from a table according to a search, such as removing all entries from a table of metabolites where the metabolite name contains “arginine”?
- **Export Table to File**: Can the portal export the contents of a table to a data file?
- **Share Table with Selected Users**: Can a user share a table with a specific set of users?
- **Share Table with the Public**: Can a user share a table with the general public?

**Table 8:** Table-based analysis capabilities.

**Table capability**	**BioCyc**	**KEGG**	**Ensembl Bacteria**	**KBase**	**IMG**	**PATRIC**
**Table datatypes:**						
Genomes	no	no	no	no	no	YES
Genes	YES	no	no	no	YES	YES^a^
Proteins	YES	no	no	no	YES	YES
RNAs	YES	no	no	no	YES	YES
Metabolites	YES	no	no	no	Partial	no
Pathways	YES	no	no	no	Partial	YES
Reactions	YES	no	no	no	Partial	no
Promoters	YES	no	no	no	no	no
Terminators	YES	no	no	no	no	no
Transcription factor binding sites	YES	no	no	no	no	no
Transcription units	YES	no	no	no	Partial	no
Publications	YES	no	no	no	no	no
Transciptomics experiments	no	no	no	no	partial	YES
Biosynthetic clusters	no	no	no	no	YES	no
Protein families	no	no	no	no	no	YES
Create table from uploaded file	YES	no	no	no	YES	YES
Create table from database query result	YES	no	no	no	YES	YES
Include database properties as table columns	YES	no	no	no	YES	YES
Create columns as computational transformations	YES	no	no	no	no	no
Set operations among tables	YES	no	no	no	YES	YES
Filter table rows	YES	no	no	no	YES	YES
Export table to file	YES	no	no	no	YES	YES
Share table with selected users	YES	no	no	no	YES	YES
Share table to the public	YES	no	no	no	no	YES

a *PATRIC provides tables of genomes and tables of features (defined sections of a genome, e.g., genes, CDS, mRNAs)*.

### 2.6. Data Content Among the Portals

Table 9 describes the types and quantities of data present in each web portal. An explanation of the rows within the Table 9 is as follows.

- **Genomes (Bact./Arch.)**: How many bacterial genomes (organisms) does the portal provide access to? Only bacteria and archaea are counted here, although some resources provide eukaryotic and viral genomes. BioCyc genomes are sourced from RefSeq, GenBank, and from the Human Microbiome Project. KEGG genomes are sourced from GenBank and RefSeq. Ensembl Bacteria genomes are sourced from the European Nucleotide Archive at the EBI, GenBank, and the DNA Database of Japan. KBase genomes are sourced from “various public sources.” IMG genomes are sourced from GenBank, RefSeq, and DOE JGI-generated data arising from their user programs. PATRIC genomes are sourced from GenBank, RefSeq, and collaborators.
- **Genome Metadata**: Does the portal contain genome metadata, such as the lifestyle of the organism, and the location of where the organism sample was obtained?
- **Regulatory Networks**: Is (gene) regulatory information provided by the site? Eleven BioCyc DBs provide regulatory networks larger than 100 transcriptional regulatory interactions.
- **Protein Localization**: Does the portal contain protein cellular locations?
- **Protein Features**: Are annotations of features of protein sequences provided by the portal? Such features include which residues bind to cofactors or to metal ions, and where signaling peptide sequences reside. IMG provides transmembrane and signal peptide features.
- **GO Terms**: Are GO term annotations provided by the site? IMG provides evidence codes for GO terms. BioCyc provides evidence terms for gene functions, pathway presence, operon presence.
- **Evidence Codes**: Are evidence codes for the annotations provided by the resource, so the level of validity of the data can be assessed?
- **Operons**: Are genes grouped into operons, where applicable?
- **Prophages**: Are potential prophages indicated on the genomes?
- **Growth Media**: Are growth media for known growth conditions of the organisms provided by the site? (BioCyc provides growth-media data for two organisms).
- **Gene Essentiality**: Are gene essentiality data under various growth conditions provided by the site? (BioCyc provides gene-essentiality data for 36 organisms).
- **Gene Clusters for Secondary Metabolites**: Does the site identify putative operons of genes encoding enzymes for the production of secondary metabolites?
- **Gene pairs with correlated expression**: Pairs of genes with correlated expression based on experimental evidence.
- **Protein-Protein interactions**: Pairs of protein with either experimental or computational evidence of interacting.
- **AMR phenotypes**: Can the site display phenotypes for antimicrobial resistance (e.g., is a strain resistant or susceptible to a particular antimicrobial compound)?

**Table 9:** Data types comparison.

**Data type**	**BioCyc**	**KEGG**	**Ensembl Bacteria**	**KBase**	**IMG**	**PATRIC**
Genomes	14,560	5,130	44,046	122,688	97,179	184,000
Bacterial genomes	14,134	4,854	43,552	121,994	66,362	181,260
Archaeal genomes	394	276	494	694	1,724	2,881
Uncultivated organisms			0		11,466	0
Genome metadata	YES	YES	no	no	YES	YES
Regulatory networks	11	no	no	no	no	no
Protein localization	YES	no	no	no	no	no
Protein features	YES	no	YES	no	Partial	YES
Protein 3-D structures	no	YES	no	no	no	no
GO terms	YES	no	YES	YES	YES	YES
Evidence codes	YES	no	no	no	YES	Partial^a^
Operons	YES	no	no	no	no	YES
Prophages	YES	no	no	no	YES	YES
Growth media	YES	no	no	YES	no	no
Gene essentiality	YES	no	no	no	no	YES
Gene clusters for secondary metabolites	no	no	no	no	YES	no
Gene pairs with correlated expression	no	no	no	no	no	YES
Protein-protein interactions	no	no	no	no	no	YES
AMR phenotypes	no	no	no	no	no	YES

a *PATRIC includes evidence codes in only two DB tables*.

### 2.7. User Experience

Table 10 contains several features that reflect the usability of the various portals. These include average loading times for typical gene pages for each portal; and other features and resources that assist the user in learning to use each portal.

- **Mean Load Time for Gene Pages**: Since gene pages are among the most commonly visited information pages within a genome web portal, the time required for the page to load in a web browser is central to the user experience. The values in this row are the average number of seconds required for each portal to load a gene page. The values are averaged across six sessions, conducted from Menlo Park, California and Richmond, Virginia to average out geographic distances to each portal. Each session tested five genes on each of the six portals. Testing was conducted using the Chrome browser version 68.0, running on MacOS 10.13.6. Testing consisted of clearing the browser cache, and pasting the URL of the gene page into the browser. The load was monitored using the ‘Network’ panel of Chrome's Developer Tools (More Tools → Developer Tools). The page was allowed to completely load (including loading large files and waiting for Ajax calls to complete). The number used is the “Finish” time in the bottom line of the panel. While some portals were disadvantaged by starting from an empty cache, forcing large files to be loaded, others were slowed by long Ajax calls. We have removed the single worst time recorded of the 30 times (5 genes × 6 sessions) for each portal.
- **Portal Information**: Lists the availability of a userguide, extensive explanatory tooltips throughout the site, recorded webinars (either downloadable files or on YouTube or similar site), and user workshops.

**Table 10:** User experience features.

**Feature**	**BioCyc**	**KEGG**	**Ensembl Bacteria**	**KBase**	**IMG**	**PATRIC**
Gene page load time (s)^a^	4.4	2.5	10.0	9.8	13.5	34.9
Tooltips	YES	no	YES	YES	YES	YES
User guide	YES	YES	YES^b^	YES	YES	YES
Webinars	YES	no	YES^b^	YES	YES	YES
Workshops	YES	?	YES	YES	YES	YES

a *The extent of gene details and visualization displayed is vastly different among sites and can lead to longer page load times. ^b^Userguide and webinars cover multiple Ensembl portals, not specifically bacteria*.

## 3. Discussion

**Figure 1:**
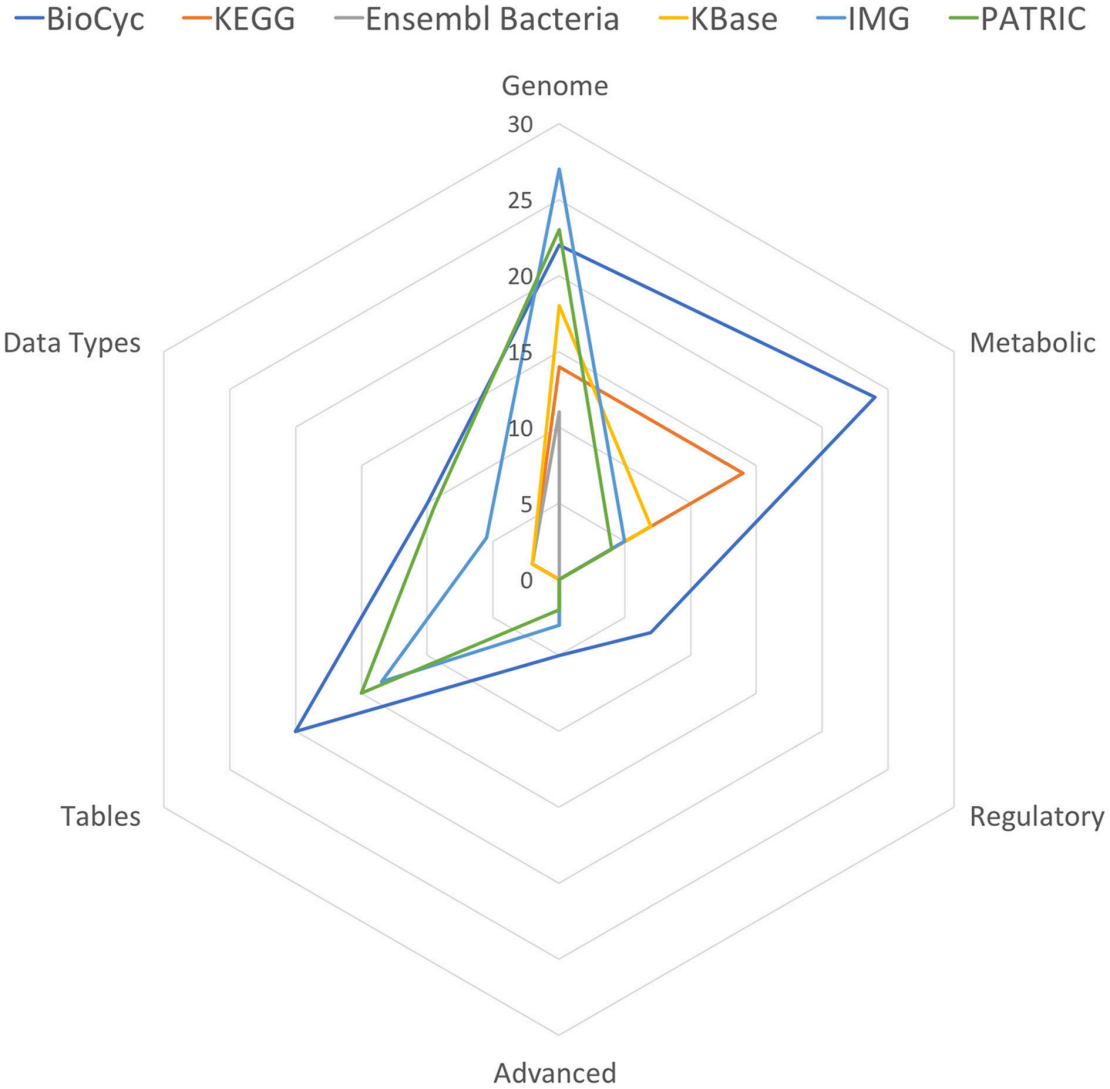
Spider plot of the data in Table 11, excluding the Multi-Search row to enhance resolution.

Table 11 summarizes the number of capabilities present in each portal. In each row of Table 11 we have summed the counts in the column for each portal from the specified tables, with each “YES” counted as 1, each “partial” counted as 1/2, and each “no” counted as 0. These data are also presented in Figure 1.

**Table 11:** Tallies of portal capabilities from previous tables.

**Tool**	**BioCyc**	**KEGG**	**Ensembl Bacteria**	**KBase**	**IMG**	**PATRIC**
Genome	22	14	11	18	27	23
Metabolic	24	14	0	7	5	4
Regulatory	7	0	0	0	0	0
Advanced	5	2	3	2.5	3	2
Tables	20	0	0	0	13.5	15
Multi-search	49	12	7	10	32	15
Data Types	10	2	2	2	5.5	9.5
Totals (excl Multi)	88	32	16	29.5	54	53.5

*Row “Genome” summarizes the major capabilities for genomics tools present in Table 1. Row “Metabolic” summarizes the major capabilities for metabolic tools present in Table 4. Row “Regulatory” summarizes the regulatory capabilities discussed in section 2.3. Row “Advanced” summarizes the major capabilities for advanced tools present in Table 7. Row “Tables” summarizes table-driven analysis capabilities for each portal present in Table 8. Row “Multi-Search” summarizes the number of multi-search capabilities for each portal present in Tables 2, 3, 5, 6. Row “Data Types” summarizes the number of datatypes provided by each portal present in Table 9, from row “Genome Metadata” downward. Row “Totals” sums each column but excludes the Multi-Search row because Multi-Search operations tend to be much smaller than operations in other categories*.

BioCyc received the highest tally (88). IMG (54) and PATRIC (53.5) were essentially tied for second. KEGG, KBase, and Ensembl Bacteria ranked fourth, fifth, and sixty with tallies of 32, 29.5, and 16, respectively.

BioCyc has the most extensive multi-search capabilities, with IMG in second place; these portals provide users with the most extensive capabilities for finding desired information.

IMG has the most genomics capabilities, with PATRIC and BioCyc second and third. Ensembl Bacteria has the fewest genomics capabilities. BioCyc and IMG have the most powerful gene/protein multi-search capabilities. BioCyc has the most extensive capabilities for DNA/RNA site multi-searches.

BioCyc has the most extensive metabolic capabilities. KEGG ranks second; it lacks metabolic modeling capabilities, and it lacks network analysis tools such as dead-end metabolite analysis and chokepoint analysis. BioCyc has the most extensive metabolic multi-search capabilities, with IMG second.

Table-analysis tools make extensive data analysis capabilities available to users that in many cases would otherwise require assistance from a programmer. BioCyc has the most extensive table-based capabilities, with PATRIC ranking second and IMG ranking third. KEGG, Ensembl Bacteria, and KBase completely lack table-based capabilities.

PATRIC has the largest number of genomes, with KBase and IMB ranked second and third, respectively; KEGG has the smallest number of genomes. Most of the PATRIC genomes were assembled from whole-genome shotgun data and thus are expected to be of lower quality—only 11,803 PATRIC bacterial genomes are complete genomes.

KEGG provides the fastest loading gene pages; BioCyc pages are the second fastest. Pages for KBase, Ensembl Bacteria, and IMG are significantly slower. PATRIC gene pages are the slowest, loading 13.96 times slower than KEGG gene pages.

BioCyc contains the most extensive analysis capabilities for metabolomics and transcriptomics data, including painting omics data onto individual pathways, multi-pathway diagrams, and zoomable metabolic maps; enrichment analysis for GO terms, regulation, and pathways; and an Omics Dashboard.

BioCyc contains extensive unique content not included in any of the other portals including regulatory network data, data on growth under different nutrient conditions, experimental gene essentiality data, reaction atom mappings (also present in KEGG), and thousands of textbook page equivalents of mini-review summaries. KEGG is particularly lacking a diverse range of datatypes, for example, KEGG lacks protein features, localization information, GO terms, and evidence codes.

## 4. Conclusions

Microbial genome web portals have a broad range of capabilities, and are quite variable in terms of what capabilities they provide. We assessed the capabilities of BioCyc, KEGG, Ensembl Bacteria, KBase, IMG, and PATRIC. BioCyc provided the most capabilities overall in terms of bioinformatics tools and breadth of data content; it also provides a level of curated data content (curated from 89,000 publications) that far exceeds that within the other sites. IMG ranked second overall, second in bioinformatics tools, and second in number of genomes. KEGG ranked third overall, PATRIC ranked fourth, KBase ranked fifth, and Ensembl Bacteria ranked sixth. IMG provided the most extensive genome-related tools, with BioCyc a close second. BioCyc provided the most extensive metabolic tools, with KEGG ranked second. Ensembl Bacteria provided no metabolic tools. PATRIC provided the largest number of genomes. BioCyc provided extensive regulatory network tools (and data) that are not present in any of the other portals. BioCyc provided the most extensive SmartTable tools and the most extensive omics data analysis tools.

## Author Contributions

PK directed the project and wrote much of the manuscript. NI, MK, NK, ML, PM, WO, SP, and RS researched the portals and contributed to the manuscript.

### Conflict of Interest Statement

The authors declare that the research was conducted in the absence of any commercial or financial relationships that could be construed as a potential conflict of interest.

## Footnotes

^1^*The BioVelo Query Language*. Available online at: https://biocyc.org/bioveloLanguage.html
